# The Effects of Fatty Acids on Inflammatory Bowel Disease: A Two-Sample Mendelian Randomization Study

**DOI:** 10.3390/nu14142883

**Published:** 2022-07-14

**Authors:** Jian He, Xiaobei Luo, Hongjie Xin, Qianwei Lai, Yuanping Zhou, Yang Bai

**Affiliations:** Department of Gastroenterology, Nanfang Hospital, Southern Medical University, Guangzhou 510515, China; smuhejian@i.smu.edu.cn (J.H.); luoxiaobei63@126.com (X.L.); xinhongjie2018@163.com (H.X.); 15625172218@163.com (Q.L.); yuanpingzhou@163.com (Y.Z.)

**Keywords:** fatty acids, inflammatory bowel disease, Crohn’s disease, ulcerative colitis, mendelian randomization

## Abstract

Inflammatory Bowel Disease (IBD) is a severe relapsing inflammation of the gastrointestinal tract. The association between fatty acids (FAs) and IBD is controversial and it remains unclear whether there is a causal relationship between them. Mendelian randomization (MR) analysis was province/state for affiliations from the same country performed to clarify the causality. Eligible single nucleotide polymorphisms were selected as instrumental variables from six Genome-wide association studies, involving 114,999 individuals in UK Biobank. The summary-level data on IBD, including Crohn’s disease (CD) and ulcerative colitis (UC), were obtained from the International Inflammatory Bowel Disease Genetics Consortium with 20,883 and 27,432 individuals involved. The primary inverse variance weighted (IVW) method as well as other supplementary analysis ones were adopted to evaluate the causal relationship between diverse FAs and IBD. The tests for heterogeneity and pleiotropy, and Leave-one-out analysis were adopted to verify the stability of the results. Omega-3 FA was found to have a causal effect on UC instead of CD. For each Standard Deviation increase in Omega-3 FA genetic levels, the risk of ulcerative colitis was found to be reduced by 39.9% by the IVW method (*p* = 1.766 × 10^−4^), by 57.8% by the MR Egger (*p* = 1.11 × 10^−2^), by 51.5% by the Weighted median estimator (*p* = 7.706 × 10^−4^), by 39% by the Maximum likelihood estimation (*p* = 3.262 × 10^−4^), and by 54.5% by the penalized weighted median estimator (*p* = 1.628 × 10^−4^). No causal relationship was found between other FAs (including total FA, saturated FA, polyunsaturated FA, monounsaturated FA and omega-6 FA) and IBD. The pleiotropic test and Leave-one-out analysis both proved the validity and reliability of these MR analyses. Omega-3 FA was observed to have a protective effect against UC, providing a new perspective on the investigation of the associations between FAs and IBD.

## 1. Introduction

Inflammatory bowel disease (IBD) is characterized by chronic relapses of gastrointestinal inflammation, including Crohn’s disease (CD) and Ulcerative colitis (UC). CD is manifested as chronic abdominal pain, diarrhea and weight loss, with the characteristics of transmural inflammation and longitudinal ulceration, which involve all segments of the gastrointestinal tract [1]. UC is manifested as a bloody diarrhea phenotype, with the characteristics of superficial mucosal inflammation, extending from the rectum to the right colon [2]. The occurrence of CD and UC are common in the western world (322 and 214 per 100,000 for CD, 505 and 214 per 100,000 for UC in Europe and the USA, respectively). IBD, especially CD, requires ceaseless drug intervention and surgery to maintain a stable condition, due to the recurrence and severity of clinical symptoms, which has a non-negligible impact on the daily life of patients, and also brings huge economic and psychological burdens to their families and even society [1,2,3].

Genetic susceptibility, environmental factors and intestinal microbiota are considered to be the three major factors in the pathogenesis of CD and UC [1,2]. Fatty acids (FAs) can regulate intestinal mucosal inflammation, which may further affect IBD occurrence [4]. The association between FAs and IBD is controversial in observational studies. Research has shown that the increased intake of saturated FAs, monounsaturated FAs, total polyunsaturated FAs, omega-3 FAs, omega-6 FAs are associated with elevated risk of CD [5,6]. However, other studies have indicated that omega-3 FAs can alleviate intestinal inflammatory and prevent CD [7,8]. In another prospective study, no association was detected among saturated FAs, monounsaturated FAs, total polyunsaturated FAs, or omega-6 FAs with IBD [9]. These confusing results might arise from incomplete elimination of bias, the uncertain measurement of dietary FAs, and the difficulty in persistent monitoring of FAs in traditional observational studies. Additionally, it is hard to conduct Randomized Controlled Trials (RCTs), due to high costs and ethical concerns.

Mendelian randomization (MR) is a statistical method, in which genetic instrumental variables (IVs) in non-experimental data are used to assess causal effects of an exposure on outcomes [10]. The MR method is a useful technique for avoiding residual confounding, preventing reverse causality, and handling situations where exposure factors are expensive or difficult to measure [10]. Instrumental variable is a measurable quantity associated with the exposure of interest instead of confounders for the outcome. It does not affect the outcome directly, but indirectly, via the hypothesized causal pathway through the exposure under investigation [10]. Instrumental variables are not limited to genetic variants; changes in government policy, geographic location, and physician prescribing preference can also be used as instrumental variables [10]. Genetic variant is an effective instrumental variable for Mendelian randomization study. Genome-wide summary association studies (GWASs), commonly referring to single nucleotide polymorphisms (SNPs), are used as IVs, because parental alleles are randomly assigned to offspring, which is equivalent to the random grouping process in RCT studies, and genetic variants from parents stay unchanged after birth, which, thus, is chronologically plausible and avoids reverse causality [10,11]. Therefore, this study aimed to perform a two-sample MR analysis to evaluate the causal relationship between diverse FAs and IBD by utilizing summary data from large-scale open-access GWASs.

## 2. Materials and Methods

### 2.1. Data Source and Open-GWAS Statistics 

The UK biobank (UKB) is a well-known repository for biomedical data and research resources, in which genetic and clinical information was collected from half a million participants in the United Kingdom [12]. The International Inflammatory Bowel Disease Genetics Consortium (IIBDGC) is a powerful organization intended to identify genetic risk factors for IBD and its related clinical characteristics, and to explore the interaction between genetic risks and the disease, as well as its phenotypes. The Consortium is a global collaborative project with researchers from more than 20 countries, involving over 75,000 IBD patients [13]. The Integrative Epidemiology Unit (IEU) OpenGWAS project is mainly comprised of publicly available datasets. These databases are now available as resources for extensive analyses, such as MR analysis [14].

According to previous studies [9], FAs were divided into total FA, saturated FA, polyunsaturated FA, and monounsaturated FA. Additionally, two individual polyunsaturated FAs, Omega-3 FA and Omega-6 FA, were examined in this study for their controversial effects on IBD in many studies. IBD was classified into CD and UC. The SNPs associated with total FA, saturated FA, polyunsaturated FA, monounsaturated FA, Omega-3 FA and Omega-6 FA were extracted from the UKB cohort. The SNPs associated with CD and UC were obtained from IIBDGC participants. IBD GWAS statistics contained 12,924 IBD European-descent patients and 35,391 European-descent controls. The proportion of males was 45.2 and 52.1 in the CD and UC cohorts, respectively. The author performed a meta-analysis of seven genome-wide CD datasets and eight genome-wide UC datasets [15]. The GWAS data were all provided by the IEU OpenGWAS database. 

Ethical approval and patient consent had already been obtained in the preliminary studies from the UKB and IIBDGC, and, thus, they were not required in this study.

### 2.2. SNPs Selection and Assumption

**Figure 1:**
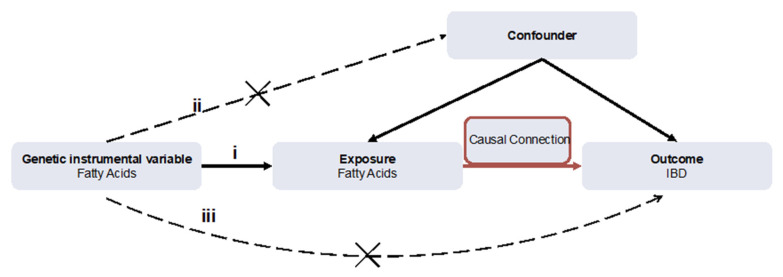
The flow diagram of the Mendelian randomization (MR) study. (i) The genetic instrumental variables (IVs) are strongly associated with fatty acids (FAs); (ii) The genetic IVs do not affect the outcome through the confounders; (iii) The genetic IVs do not affect inflammatory bowel disease (IBD) directly, but only via indirect exposure.

As shown in Figure 1, if a genetic variant can be applied to the estimation of a causal effect, the three following core instrumental variable assumptions must be satisfied: (i) the variant is strongly associated with the exposure (correlation hypothesis); (ii) the variant affects no outcome through the confounders (independence hypothesis); (iii) the variant directly affects no outcome, only via indirect exposure (exclusion hypothesis) [10]. The following criteria were set for SNPs selection. In order to satisfy assumption (i), a statistical significance level (*p* < 5 × 10^−8^) was strictly set to satisfy genome-wide significant associations. A threshold (R^2^ < 0.001) and specific mutation frequency, Minor Allele Frequency (MAF ≥ 1%), were set for SNPs to attenuate linkage disequilibrium (LD). In order to satisfy assumptions (ii) and (iii), each SNP was checked at *PhenoScanner*, an open database of human genotype–phenotype associations, and Genome-wide SNPs significantly associated with the potential confounders and outcomes were eliminated. For variant harmonization, the palindromic variants were excluded, because it was difficult to verify their correctly orientated alleles.

### 2.3. Statistical Analysis of Primary MR 

The inverse-variance weighted (IVW) method was adopted as the primary analysis method, because it was considered to be the most efficient analysis with valid instrumental variables [16]. When the pleiotropic effects of IVs were absent and the sample size was large enough, the IVW estimate was consistent, efficient and close to the true value [17]. The multiplicative random effect IVW model was applied when heterogeneity was statistically significant (*p* < 0.05). Otherwise, the fixed effects model was adopted.

### 2.4. Supplementary and Sensitivity Analysis

In addition to IVW, other robust methods for MR were used to ensure the consistency and efficiency of the results. The Maximum likelihood method was used to estimate probability distribution parameters by maximizing the likelihood function with low standard errors [18]. The MR Egger method was used to test directional pleiotropy and causal effects, and to estimate the causal effects under a weaker assumption, the InSIDE (Instrument Strength Independent of Direct Effect) assumption [19]. The penalized weighted median estimator was a new analysis method for modifying standard weighted median MR, by which the weight was further put on the instruments, and any instrument that substantially contributed to the heterogeneity statistics was penalized [20,21]. The estimate was validly calculated via Weighted Medians when over half of the selected SNPs were valid genetic variants [17]. Scatter, forest, and funnel plots were used to visualize the results and showed the efficiency and stability of the MR study.

In order to verify the conformity of each SNP, the heterogeneity test was performed by means of the MR Egger and IVW methods to calculate Cochran Q statistics and find the heterogeneity among genetic variants. The heterogeneity was statistically significant (*p* < 0.05). Pleiotropy refers to a genetic variant with more than one independent phenotypic effect, which may affect causal pathways [10]. The MR Egger intercept test was applied to the assessment for the horizontal pleiotropy. Leave-one-out analysis was performed by omitting the genetic variants one by one, and MR analysis was still conducted on the rest. The causal relationship would be credible and stable if the result of the leave-one-out analysis conformed to that of the global IVW analysis. 

All analyses were performed by R (version 4.1.1) and the TwoSampleMR package.

## 3. Results

### 3.1. IIBDGC GWASs of FAs

The GWASs of diverse FAs were all obtained from UK biobank, involving 114,999 participants and 55–66 FA strongly related SNPs were extracted. The GWASs of CD and UC were obtained from IIBDGC with 20,883 and 27,432 participants involved, respectively (Table 1).

In order to find out the effect of FA-related genetic IVs on CD and UC, robust genetic IVs in six types of FAs were identified in the IIBDGC database through open GWAS platforms (gwas.mrcieu.ac.uk). The identification process was conducted by using the functions of “extract_outcome_data” and “harmonise_data” and 14–23 SNPs were extracted for the subsequent causality analysis (Table 2 and Table 3).

### 3.2. Primary MR Analysis

**Figure 2:**
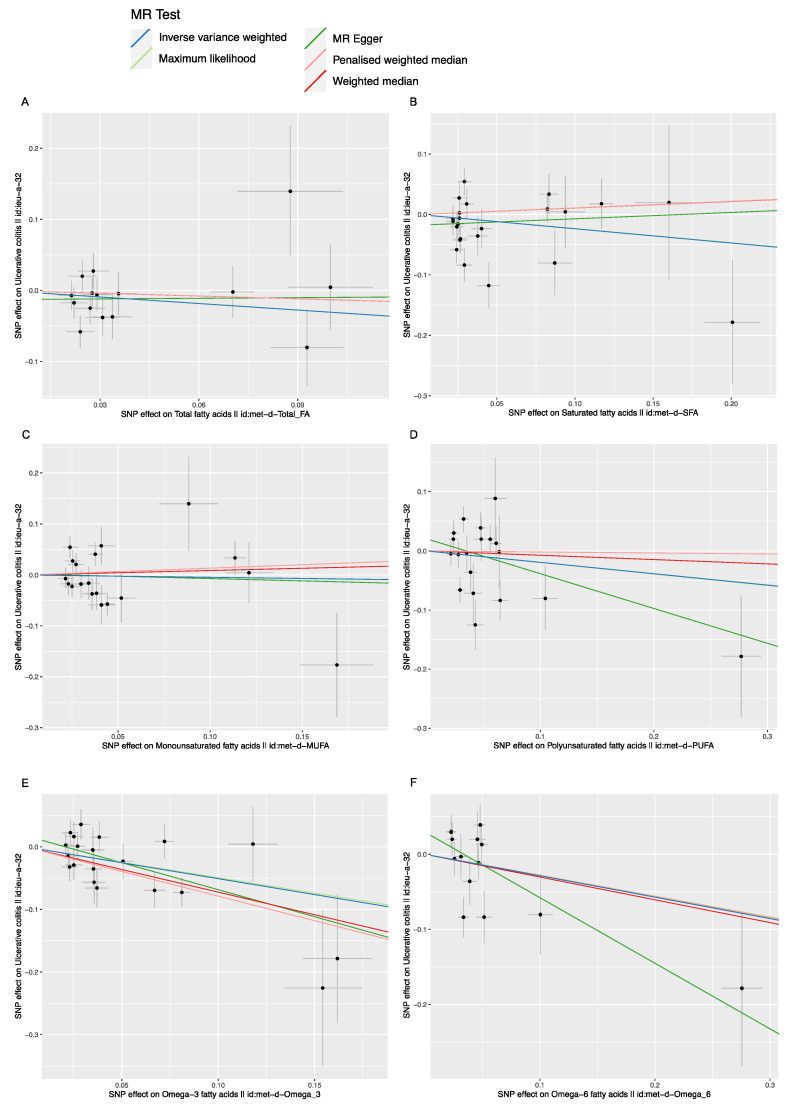
The scatter plot of fatty acids (FAs) and Ulcerative colitis (UC), *X*-axis, the single nucleotide polymorphism (SNP) effect and standard errors (SEs) on each of the selected SNPs from FA genome-wide summary association study (GWA) dataset *Y*-axis, the SNP effect and SEs on UC from Crohn’s disease (CD) Genome-wide summary association study (GWA) datasets. (**A**) Analysis of Total FA and UC; (**B**) Analysis of saturated FA and UC; (**C**) Analysis of monounsaturated FA and UC; (**D**) Analysis of polyunsaturated FA and UC; (**E**) Analysis of Omega-3 FA and UC; (**F**) Analysis of Omega-6 FA and UC.

As shown in Table 2 and Table 3, no causal relationship was detected between the FAs (including total FA, saturated FA, polyunsaturated FA, monounsaturated FA, Omega-6 FA) and the IBD (including CD and UC), with *p*-values (≥0.05) measured by the IVW method. No causal relationship was found between Omega-3 FA and CD with *p*-value (0.776) measured by the IVW method. However, a negative causal relationship was found between Omega-3 FA and UC [IVW, OR/95%CI: 0.601/(0.461, 0.784), *p* (1.766 × 10^−4^)], as shown in Table 3 and Figure 2E. For each Standard Deviation (SD) increase in genetically determined Omega-3 FA levels, the risk of UC was found to be reduced by 39.9%, according to the IVW method.

### 3.3. Supplementary and Sensitivity Analysis

In addition to the primary IVW analysis method, other statistical methods, including the MR Egger, weighted median estimator, maximum likelihood estimation and penalized weighted median estimator methods were adopted to verify the accuracy of the main results. These supplementary analysis methods were used to confirm the results that the FAs (including total FA, saturated FA, polyunsaturated FA, monounsaturated FA, and Omega-6 FA) had no causal effect on CD and UC. No causal relationship was found between the Omega-3 FA and CD (*p =* 0.701, 0.566, 0.648, 0.091 respectively) (Table 2 and Table 3). A negative causal relationship between Omega-3 FA and UC was detected by these supplementary MR methods (*p =* 0.011, 7.706 × 10^−4^, 3.262 × 10^−4^, 1.628 × 10^−4^, respectively). For each SD increase in genetically determined Omega-3 FA levels, the risk of ulcerative colitis was found to be reduced by 57.8%, 51.5%, 39.0% and 54.5% by the MR Egger method, weighted median estimator, maximum likelihood estimation and penalized weighted median estimator, respectively.

**Figure 3:**
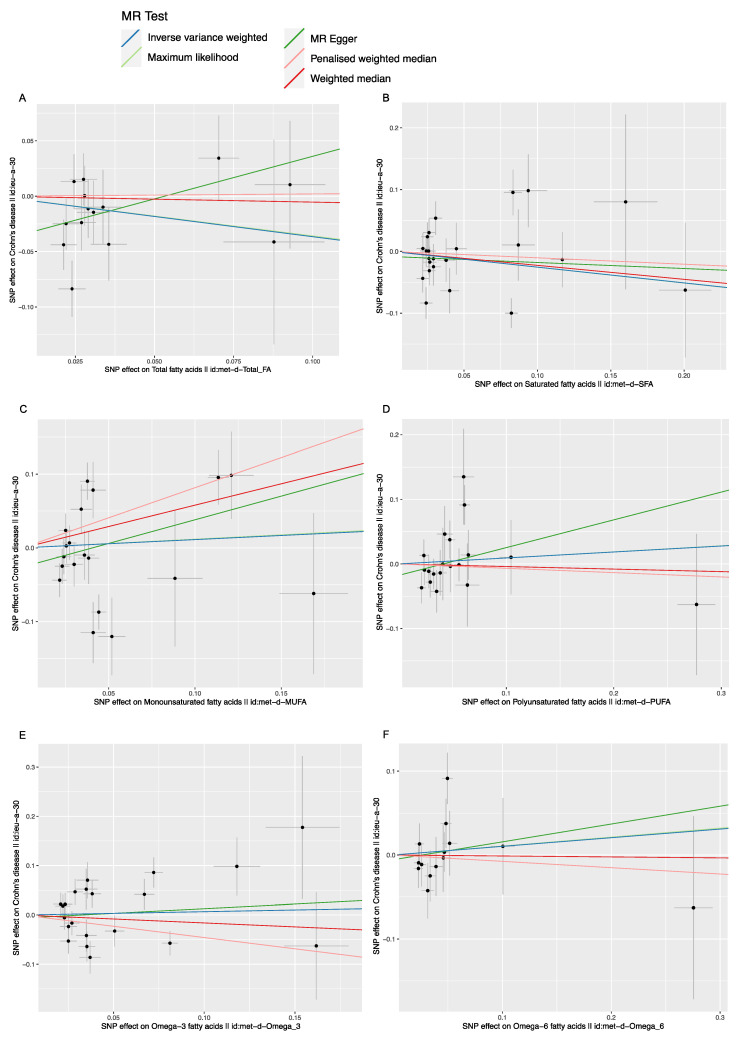
The scatter plot of fatty acids (FAs) and Crohn’s disease (CD). *X* axis, the single nucleotide polymorphism (SNP) effect and SEs (standard errors) on each of the selected SNPs from FA Genome-wide summary association study (GWA) datasets. *Y* axis, the SNP effect and SEs on CD from CD Genome-wide summary association study (GWA) datasets. (**A**) Analysis of Total FA and CD; (**B**) Analysis of saturated FA and CD; (**C**) Analysis of monounsaturated FA and CD; (**D**) Analysis of polyunsaturated FA and CD; (**E**) Analysis of Omega-3 FA and CD; (**F**) Analysis of Omega-6 FA and CD.

The scatter plot was used to visualize the effect size of each MR method (Figure 2 and Figure 3). The forest plot was applied to the visualization of the individual SNP estimates of outcomes (Figures S1 and S2). The funnel plot was adopted to show the distribution balance of single SNP effects (Figures S3 and S4). From these plots, it could be concluded that the effect of each SNP, and its distribution, were in equilibrium.

The heterogeneity was measured by the Cochran’s Q statistic. As shown in Table 4, the heterogeneity analysis results showed that significant statistical heterogeneity was detected among the genetic instrumental variables in the effects of saturated FA (IVW, *p* = 2.94 × 10^−3^), monounsaturated FA (IVW, *p* = 7.56 × 10^−7^) and Omega-3 FA (IVW, *p* = 1.12×10^−4^) on CD, and, also, those of saturated FA (IVW, *p* = 1.41 × 10^−3^), polyunsaturated FA (IVW, *p =* 4.33 × 10^−4^), monounsaturated FA (IVW, *p* = 7.78 × 10^−3^) and Omega-6 FA (IVW, *p =* 1.27 × 10^−2^) on UC. Therefore, the multiplicative random effects IVW model was applied in these associations to calculate the causal effects. In addition, no significant statistical heterogeneity was found among the genetic instrumental variables in the effects of total FA (IVW, *p =* 0.155), polyunsaturated FA (IVW, *p* = 0.226) and Omega-6 FA (IVW, *p* = 0.343) on CD, and also those of total FA (IVW, *p* = 0.273) and Omega-3 FA (IVW, *p* = 0.149) on UC. Therefore, the fixed effect IVW model was used for the primary MR analysis.

**Figure 4:**
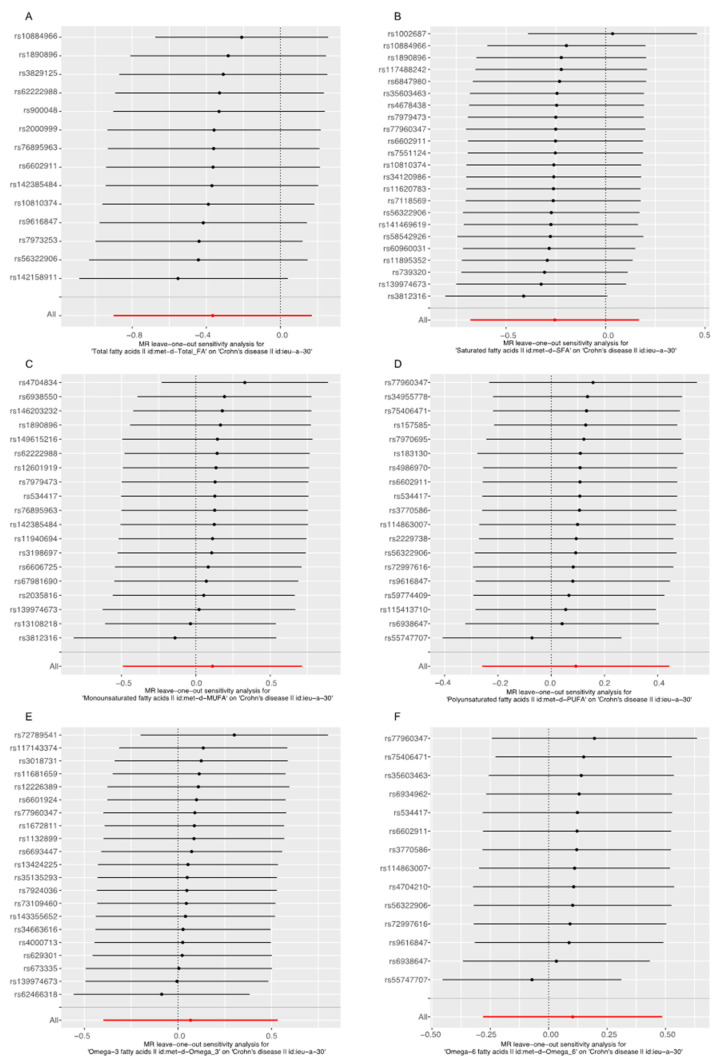
Leave-one-out analysis for the effect of fatty acids (FAs) and Crohn’s disease (CD). (**A**) Analysis of Total FA and CD; (**B**) Analysis of saturated FA and CD; (**C**) Analysis of monounsaturated FA and CD; (**D**) Analysis of polyunsaturated FA and CD; (**E**) Analysis of Omega-3 FA and CD; (**F**) Analysis of Omega-6 FA and CD.

**Figure 5:**
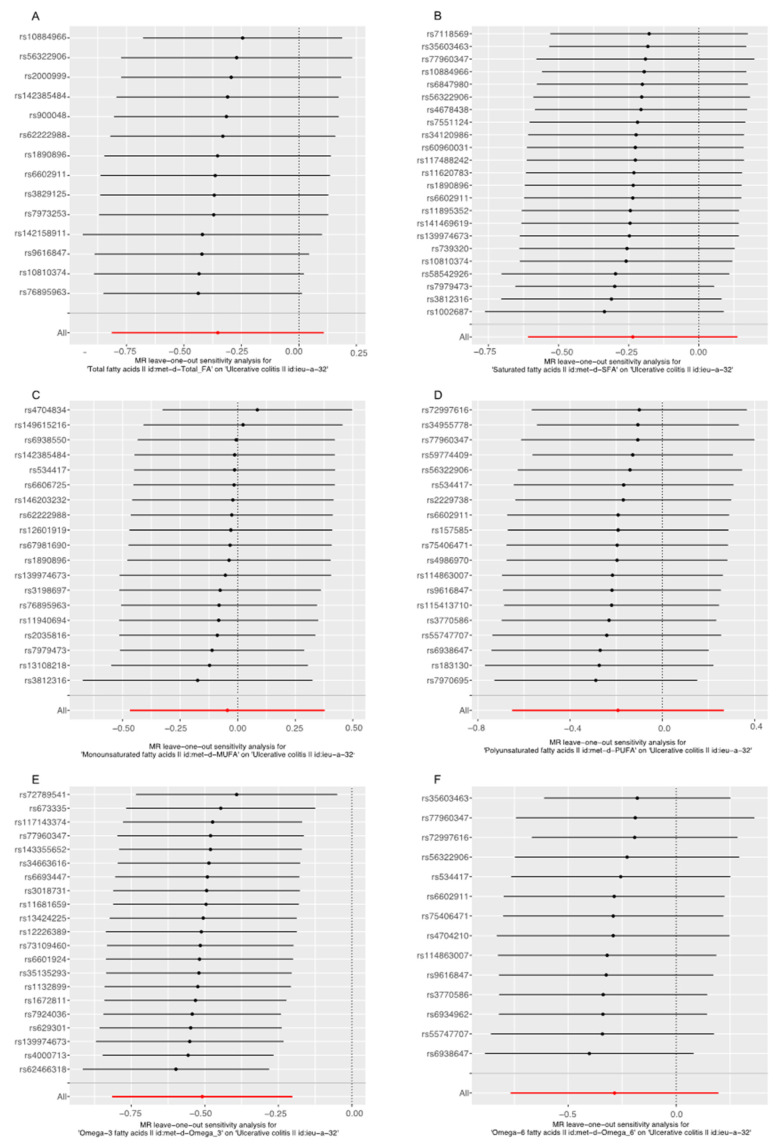
Leave-one-out analysis for the effect of fatty acids (FAs) and Ulcerative colitis (UC). (**A**) Analysis of Total FA and UC; (**B**) Analysis of saturated FA and UC; (**C**) Analysis of monounsaturated FA and UC; (**D**) Analysis of polyunsaturated FA and UC; (**E**) Analysis of Omega-3 FA and UC; (**F**) Analysis of Omega-6 FA and UC.

The leave-one-out analysis was performed to evaluate the single SNP effect on the final MR result. As shown in Figure 4 and Figure 5, after omitting the single SNP sequentially, the remaining causal effects of diverse FAs on CD and UC found in the leave-one-out analysis were consistent with that found in the primary MR studies, which showed no single SNP that played a significant role in the final result, suggesting the MR studies were robust, stable and reliable.

The test for horizontal pleiotropic effects was performed to determine whether FAs-related genetic instrumental variants could lead to IBD through other potential pathways. As shown in Table 5, no significant horizontal pleiotropy was found in our MR analyses (all *p* values ≥0.05), which indicated that these MR studies were hardly likely to be affected by potential confounding pathways, and the results were robust and reliable.

## 4. Discussion

Epidemiological evidence has suggested that different FAs play a diverse role in the onset of IBD, but their exact roles have remained, as yet, unclear [6,22,23]. It was found in this study that Omega–3 FA had a negative causal effect on UC instead of CD, and that no causal relationship was detected between the FAs (including total FA, saturated FA, monounsaturated FA, polyunsaturated FA and Omega–6 FA) and IBD. 

There were fifty-two genetic IVs obtained from UK biobank strongly associated with Omega-3 FA. Only twenty-two SNPs were extracted from IIBDGC in the effect analysis of UC, after eliminating potential confounders and other IBD-related characteristics, such as suffering from depression, being a worrier, cholesterol issues, smoking, taking contraceptives and having sclerosing cholangitis. Apart from the primary IVW analysis, the other four MR analysis methods, including the MR Egger, weighted median estimator, maximum likelihood estimation, penalized weighted median estimator, also confirmed that increased Omega-3 FA level caused by genetic factors can reduce the risk of UC. No significant pleiotropic effect between Omega-3 FA genetic variants and UC was detected in the pleiotropic analysis (Table 2) and no statistically significant single SNP associated with the result was detected in the leave-one-out analysis (Figure 3E). These results demonstrated that Omega-3 FA genetic variants could affect the onset of UC via Omega-3 FA instead of other pathways. Therefore, it could be concluded that there existed a causal relationship between Omega-3 FA and UC. No causal relationship was detected in the remaining groups in this study. The sensitive analysis, including the pleiotropic test and the leave-one-out analysis, confirmed the robustness and reliability of the relationship. Supplementary MR methods also proved the validity of the results. Among the twelve MR analysis groups, significant statistical heterogeneity was detected in seven groups. The heterogeneity might come from such factors as age and education. The exact source of heterogeneity remained unknown, due to the limitation of original data access. The multiplicative random effect IVW model was used to alleviate this influence. An IV could be understood as an exogenous variable associated with endogenous exposure, which was used to estimate the mean difference in outcomes under different average values of exposure when all other factors were, on average, equal. Under the assumption of consistency, this unproven estimate could be interpreted as a causal effect. The consistency assumption suggests that when the exposure value is observed to take a certain value, the outcome value obtained is the same as the one obtained when the exposure value is set to take the same value [10]. Exploring the causal effect of fatty acids on IBD was the key point in the investigation. Genetic variants were only instrumental variables to perform the analysis. It is difficult to accurately detect and monitor the intake value of FAs, so it is difficult to provide the value of FAs, which is a shortcoming of a traditional epidemiological study, but a strongpoint of a Mendelian randomization analysis. In a traditional epidemiological study, dietary and other habits could have enormous impacts on the relation of FA with IBD. The original article of FA GWAS statistics was not available, and the influence of dietary and other habits on FAs levels was hardly judged. However, our study explored the IBD risk variation based on FA level determined by genetic variants. The MR analysis was designed to avoid traditional confounders, such as dietary and other habits, by introducing instrumental variables, and to monitor their interference by sensitivity analysis. So, the influences of dietary and other habits could be balanced and would not affect the results.

The effect of FAs, as a major component of the western diet, on IBD has been widely investigated. In a systematic review [6], involving 1269 CD and 1340 UC individuals, as well as over 4000 controls, it was concluded that saturated FA, monounsaturated FA, polyunsaturated FA, omega-3 and omega-6 FA could increase the risk of CD, while total FA, polyunsaturated FA, omega-6 FA could increase the risk of UC. In a case control study [5] involving 182 pediatric CD patients and 250 controls, it was found that individuals with higher omega-6/omega-3 ratios are more susceptible to the development of CD. The result of this MR study was consistent with that of the research conducted by Ananthakrishnan, A.N. et al. [9]. By exploration of the relationship between FAs (including total FA, monounsaturated FA, saturated FA, polyunsaturated FA, omega-3 and omega-6 FA) and IBD in a prospective cohort, involving 170 805 women with a follow-up lasting over 26 years, it was found that long-chain omega-3FAs might be associated with a reduced risk of UC. In this MR study, the weaknesses of traditional observational studies were overcome by utilizing a new statistical approach and more reliable conclusions with a large sample size were drawn.

The FAs, especially omega 3 and omega 6 FAs, play a great role in autoimmune disease and immune cell metabolism [24,25,26,27]. Omega 6 FA has been considered to be a pro-inflammatory factor, while omega 3 FA has been considered to be an anti-inflammatory compound. The intestinal microbiota can exert a complicated and significant effect on the association between FAs and intestine-related disorders [24,27]. However, the association between FAs and IBD has always been controversial [6,22].

There exist several underlying mechanisms accounting for the association of omega 3 FA with a reduced risk of UC. Firstly, an elevated omega 3 FA level can increase the amount of Eicosapentaenoic acid (EPA) and docosahexaenoic acid (DHA), and decrease the level of arachidonic acid (ARA), which can alter the compounds of membrane phospholipids and further modulate the formation of lipid rafts to adapt to inflammatory stimulation [28,29,30]. Meanwhile, elevated EPA can inhabit the function of ARA and decrease the expression of COX-2-related genes, which have an anti-inflammatory effect [28]. Secondly, omega-3 FA can promote NFκB activation, through the activation of the PPAR-γ receptor, which can inhibit the generation of inflammatory cytokines, such as TNF and IL-6 [31,32,33].

The strengths of this MR study are listed as follows. Firstly, the design of the research was based on three principal instrumental variable assumptions and conformed to the Checklist for performing MR investigations [34]. Thus, the conclusions drawn in this study were reasonable and could be trusted. Secondly, the two large-scale GWASs were both obtained from European ancestries, which allowed the bias of population stratification to be avoided. Thirdly, a total of five MR analysis methods were applied to the evaluation of the consistency of causal effects.

Meanwhile, some weaknesses cannot be ignored in this study. Firstly, both UKB and IIBDGC participants were Europeans, and, thus, the generalizability of the results was limited. Secondly, the IBD patients were from different medical centers, and the differences in diagnosis methods, information acquisition and data processing might bias the results. Thirdly, this study only provided robust and reliable evidence for the effect of different types of FAs on IBD risk. The effect of FAs on established IBD patients has not yet been explored. Fourthly, the biological mechanism and genetic co-inheritance, such as gene expression, gene-gene interaction, gene–environment interaction and potentially epigenetic factors, are potential factors that may violate instrumental variables assumptions and affect result accuracy [10]. Therefore, in instrumental variable selection and processing, methods, such as removing linkage disequilibrium and detecting pleiotropy, were adopted to minimize and monitor their impact on the results. However, the effect could not be completely eliminated. Fifthly, in the original literature for genetic variants, the author did not provide the cohort follow-up time. So, though we could not judge the influence of the follow-up time, it might bring bias to the results.

This MR study indicated that omega-3 FA was a significant protective factor of UC instead of CD. Total FA, Saturated FA, Polyunsaturated FA, Monounsaturated FA, Omega-6 FA were not associated with the risk of CD and UC. However, our study was based on FA level determined by genetic variants, which could only account for part of the IBD risk variation. So, a large-sample random control cohort study is still required to clarify the association between FAs and IBD. Meanwhile, more types of FAs should be explored to determine their causal effects on IBD. Our MR study overturned the traditional view that pro-inflammatory FAs may be the risk factors for IBD, and confirmed that omega-3 FA is a protective factor of UC.

## 5. Conclusions

Our MR study showed that Omega–3 FA had a negative causal effect on UC instead of CD. Total FA, Saturated FA, Polyunsaturated FA, Monounsaturated FA, Omega-6 FA had no association with the risk of CD and UC.

## Figures and Tables

**Table 1:** The list of Genome-wide summary association studies (GWAs) included in the Mendelian randomization (MR) study.

Exposures	GWAS ID	Consortium	Sample Size	No. of Strongly Related SNPs	Adjustment	Population
Total FA	met-d-Total_FA	UK biobank	114,999	64	Depression, Worrier, Cholesterol, Smoking, Contraceptives and sclerosing cholangitis	European
Saturated FA	met-d-SFA	UK biobank	114,999	55		
Monounsaturated FA	met-d-MUFA	UK biobank	114,999	66		
Polyunsaturated FA	met-d-PUFA	UK biobank	114,999	66		
Omega-3 FA	met-d-Omega_3	UK biobank	114,999	52		
Omega-6 FA	met-d-Omega_6	UK biobank	114,999	63		
Outcomes	GWAS ID	Consortium	Cases	Control	Population	
Crohn’s disease	ieu-a-30	IIBDGC	5956	14,927	European	
Ulcerative colitis	ieu-a-32	IIBDGC	6968	20,464		

**Table 2:** MR analysis results of diverse fatty acids (FAs) and Crohn’s disease (CD).

Fat Acids (Exposure)	MR Method	No. SNP	β	SE	OR(95%CI)	*p*
total FA	MR Egger	14	0.769	0.595	2.158 (0.672, 6.926)	0.220
	Weighted median		−0.053	0.316	0.948 (0.510, 1.762)	0.867
	IVW(fixed effects)		−0.366	0.232	0.694 (0.441, 1.092)	0.114
	Maximum likelihood		−0.359	0.235	0.697 (0.440, 1.108)	0.128
	Penalised weighted median		0.021	0.315	1.020 (0.550, 1.896)	0.948
saturated FA	MR Egger	23	−0.097	0.394	0.907 (0.419, 1.964)	0.807
	Weighted median		−0.226	0.246	0.797 (0.491, 1.295)	0.356
	IVW(random effects)		−0.255	0.217	0.775 (0.507, 1.184)	0.238
	Maximum likelihood		−0.257	0.143	0.774 (0.585, 1.024)	0.072
	Penalised weighted median		−0.104	0.261	0.901 (0.541, 1.501)	0.689
polyunsaturated FA	MR Egger	19	0.430	0.355	1.537 (0.766, 3.085)	0.243
	Weighted median		−0.039	0.231	0.961 (0.612, 1.510)	0.864
	IVW(fixed effects)		0.093	0.162	1.097 (0.799, 1.506)	0.567
	Maximum likelihood		0.094	0.163	1.098 (0.798, 1.512)	0.565
	Penalised weighted median		−0.067	0.234	0.935 (0.591, 1.480)	0.775
monounsaturated FA	MR Egger	19	0.641	0.584	1.898 (0.604, 5.970)	0.288
	Weighted median		0.578	0.263	1.784 (1.066, 2.987)	0.027
	IVW(random effects)		0.112	0.308	1.118 (0.612, 2.043)	0.716
	Maximum likelihood		0.118	0.169	1.125 (0.808, 1.568)	0.485
	Penalised weighted median		0.815	0.264	2.259 (1.346, 3.793)	0.71
Omega-3 FA	MR Egger	21	0.188	0.483	1.207 (0.468, 3.111)	0.701
	Weighted median		−0.161	0.281	0.851 (0.491, 1.476)	0.566
	IVW(random effects)		0.0675	0.238	1.070 (0.671, 1.705)	0.776
	Maximum likelihood		0.069	0.150	1.071 (0.798, 1.436)	0.648
	Penalised weighted median		−0.457	0.270	0.633 (0.373, 1.075)	0.0905
Omega-6 FA	MR Egger	14	0.213	0.388	1.237 (0.578, 2.648)	0.594
	Weighted median		−0.011	0.265	0.989 (0.588, 1.662)	0.966
	IVW(fixed effects)		0.103	0.186	1.108 (0.770, 1.595)	0.580
	Maximum likelihood		0.107	0.187	1.112 (0.771, 1.605)	0.569
	Penalised weighted median		−0.076	0.245	0.927 (0.574, 1.499)	0.758

No. SNP, number of SNPs included in the analysis. β: the regression coefficient based on fatty acid raising effect allele. SE, standard error. *p* < 0.05 represents the causal link of fatty acid with CD.

**Table 3:** MR analysis results of diverse fatty acids (FAs) and Ulcerative colitis (UC).

Fat Acids (Exposure)	MR Method	No. SNP	β	SE	OR(95%CI)	*p*
total FA	MR Egger	14	−0.051	0.602	0.951 (0.292, 3.091)	0.934
	Weighted median		−0.196	0.294	0.822 (0.462, 1.462)	0.505
	IVW(fixed effects)		−0.353	0.215	0.703 (0.461, 1.071)	0.101
	Maximum likelihood		−0.355	0.218	0.701 (0.457, 1.075)	0.103
	Penalised weighted median		−0.189	0.306	0.827 (0.455, 1.506)	0.535
saturated FA	MR Egger	23	−0.105	0.339	1.111 (0.572, 2.157)	0.759
	Weighted median		0.108	0.200	1.114 (0.758, 1.637)	0.590
	IVW(random effects)		−0.236	0.191	0.790 (0.544, 1.148)	0.216
	Maximum likelihood		−0.234	0.132	0.791 (0.611, 1.025)	0.076
	Penalised weighted median		0.109	0.192	1.115 (0.749, 1.659)	0.517
polyunsaturated FA	MR Egger	19	−0.589	0.472	0.555 (0.220, 1.401)	0.230
	Weighted median		−0.073	0.240	0.930 (0.581, 1.488)	0.761
	IVW(random effects)		−0.193	0.234	0.825 (0.521, 1.305)	0.411
	Maximum likelihood		−0.194	0.151	0.823 (0.613, 1.107)	0.199
	Penalised weighted median		−0.017	0.242	0.983 (0.611, 1.582)	0.944
monounsaturated FA	MR Egger	19	−0.091	0.425	0.913 (0.397, 2.098)	0.832
	Weighted median		0.088	0.232	1.092 (0.692, 1.723)	0.705
	IVW(random effects)		−0.045	0.216	0.956 (0.626, 1.460)	0.835
	Maximum likelihood		−0.046	0.156	0.955 (0.703, 1.295)	0.765
	Penalised weighted median		0.134	0.239	1.144 (0.717, 1.826)	0.573
Omega-3 FA	MR Egger	21	−0.863	0.307	0.422 (0.231, 0.770)	0.0111
	Weighted median		−0.723	0.215	0.485 (0.318, 0.740)	7.706 × 10^−4^
	IVW(fixed effects)		−0.509	0.136	0.601 (0.461, 0.784)	1.766 × 10^−4^
	Maximum likelihood		−0.493	0.137	0.610 (0.466, 0.799)	3.262 × 10^−4^
	Penalised weighted median		−0.787	0.209	0.455 (0.302, 0.685)	1.628 × 10^−4^
Omega-6 FA	MR Egger	14	−0.874	0.456	0.417 (0.171, 1.021)	0.079
	Weighted median		−0.303	0.249	0.738 (0.454, 1.202)	0.223
	IVW(random effects)		−0.285	0.246	0.752 (0.464, 1.217)	0.246
	Maximum likelihood		−0.278	0.173	0.757 (0.539, 1.063)	0.108
	Penalised weighted median		−0.280	0.263	0.756 (0.451, 1.265)	0.286

No. SNP, number of SNPs included in the analysis. β: the regression coefficient based on fatty acids raising effect allele. SE, standard error. *p* < 0.05 represents the causal link of fatty acid with CD.

**Table 4:** The heterogeneity test of diverse fatty acids (FAs) genetic variants in inflammatory bowel disease (IBD) Genome-wide summary association study (GWAS) datasets.

Traits (Outcome)	Fat Acids (Exposure)	Methods	Q	Q-dif	*p*
CD	total FA	MR Egger	13.249	12	0.351
		IVW	18.072	13	0.155
	saturated FA	MR Egger	51.622	21	2.160 × 10^−4^
		IVW	52.196	22	2.937 × 10^−3^
	polyunsaturated FA	MR Egger	20.667	17	0.242
		IVW	22.130	18	0.226
	monounsaturated FA	MR Egger	58.748	17	1.688 × 10^−6^
		IVW	62.658	18	7.558 × 10^−7^
	Omega-3 FA	MR Egger	51.832	19	7.012 × 10^−5^
		IVW	52.060	20	1.116 × 10^−4^
	Omega-6 FA	MR Egger	14.321	12	0.281
		IVW	14.452	13	0.343
UC	total FA	MR Egger	15.198	12	0.231
		IVW	15.579	13	0.273
	saturated FA	MR Egger	44.057	21	2.299 × 10^−3^
		IVW	47.138	22	1.408 × 10^−3^
	polyunsaturated FA	MR Egger	42.533	17	5.614 × 10^−4^
		IVW	44.869	18	4.328 × 10^−4^
	monounsaturated FA	MR Egger	35.630	17	5.13 × 10^−3^
		IVW	35.670	18	7.78 × 10^−3^
	Omega-3 FA	MR Egger	24.271	19	0.186
		IVW	26.541	20	0.149
	Omega-6 FA	MR Egger	22.678	12	0.031
		IVW	26.945	13	0.013

*p* < 0.05 is set as the significant threshold.

**Table 5:** The pleiotropic test of FAs genetic variants in inflammatory bowel disease (IBD) Genome-wide summary association study (GWAS) datasets.

Traits (Outcome)	Fat Acids (Exposure)	Egger_Intercept	se	*p*
CD	total FA	−0.041	0.020	0.059
	saturated FA	−8.271 × 10^3^	0.017	0.634
	polyunsaturated FA	−0.018	0.016	0.288
	monounsaturated FA	−0.026	0.025	0.302
	Omega-3 FA	−6.115 × 10^3^	0.021	0.775
	Omega-6 FA	−5.612 × 10^3^	0.017	0.746
UC	total FA	−0.011	0.020	0.594
	saturated FA	−0.018	0.015	0.239
	polyunsaturated FA	0.020	0.021	0.347
	monounsaturated FA	2.28 × 10^3^	0.018	0.899
	Omega-3 FA	0.018	0.013	0.198
	Omega-6 FA	0.030	0.020	0.159

*p* < 0.05 is set as the significant threshold, se standard error.

## Data Availability

All data mentioned in the manuscript are available in the website provided in the article.

## Supplementary Materials

The following supporting information can be downloaded at: https://www.mdpi.com/article/10.3390/nu14142883/s1, Figure S1. Forest plot of fatty acids and the risk of Crohn’s disease. Figure S2. Forest plot of fatty acids and the risk of Ulcerative colitis. Figure S3. Funnel plot of the inverse variance weighted MR estimate of each fatty acids SNP with Crohn’s disease versus 1/SEIV. Figure S4, Funnel plot of the inverse variance weighted MR estimate of each fatty acids SNP with Ulcerative colitis versus 1/SEIV.
