# Supplementary material for: The Effects of Fatty Acids on Inflammatory Bowel Disease: A Two-Sample Mendelian Randomization Study
Source: Nutrients. 2022 Jul 14;14(14):2883. doi: 10.3390/nu14142883 (PMC9320846; doi:10.3390/nu14142883)
Supplement: Supplementary file 1 [file nutrients-14-02883-s001.zip › nutrients-1786757-supplementary.pdf]

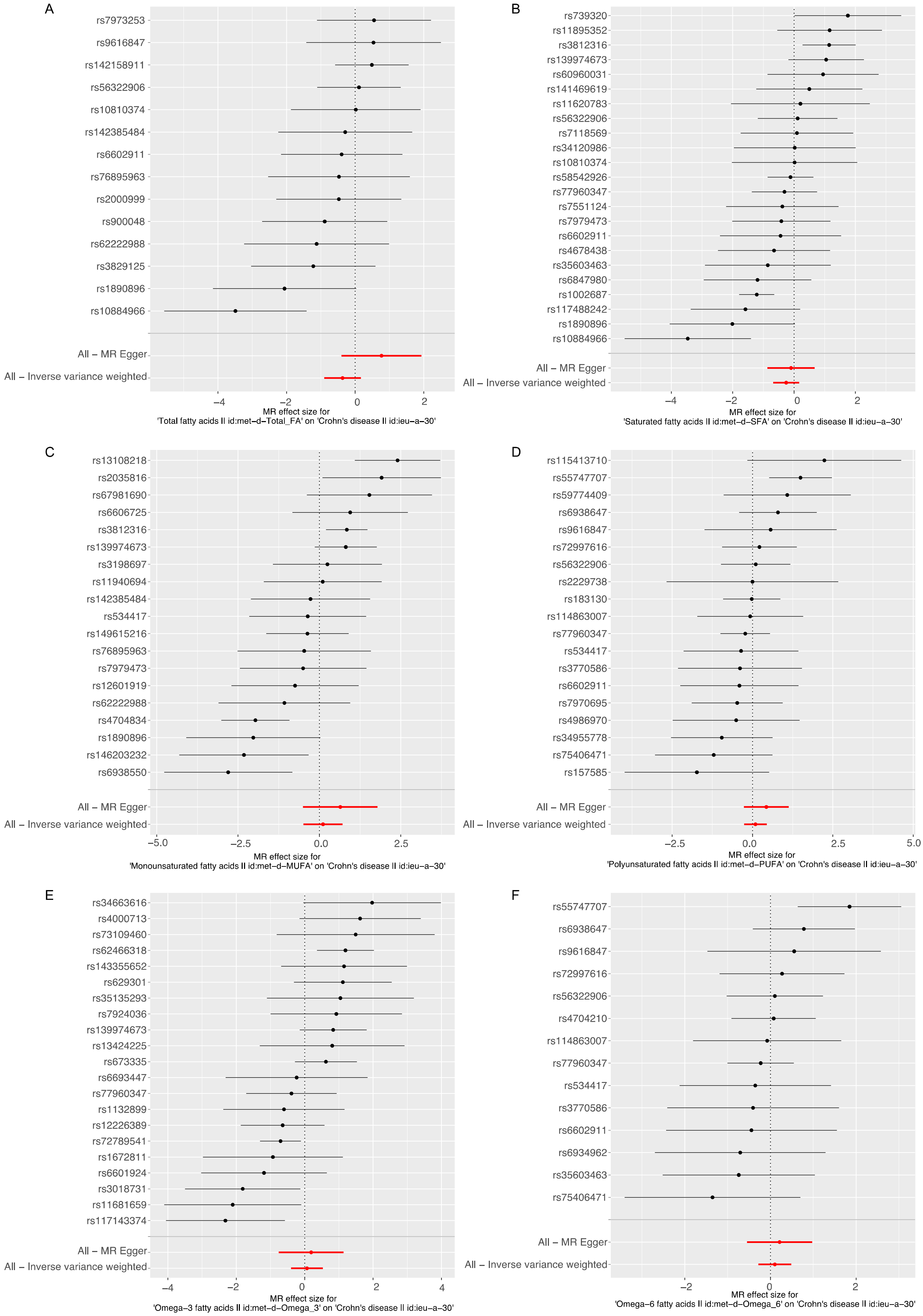

Figure S1. Forest plot of fatty acids and the risk of Crohn's disease.

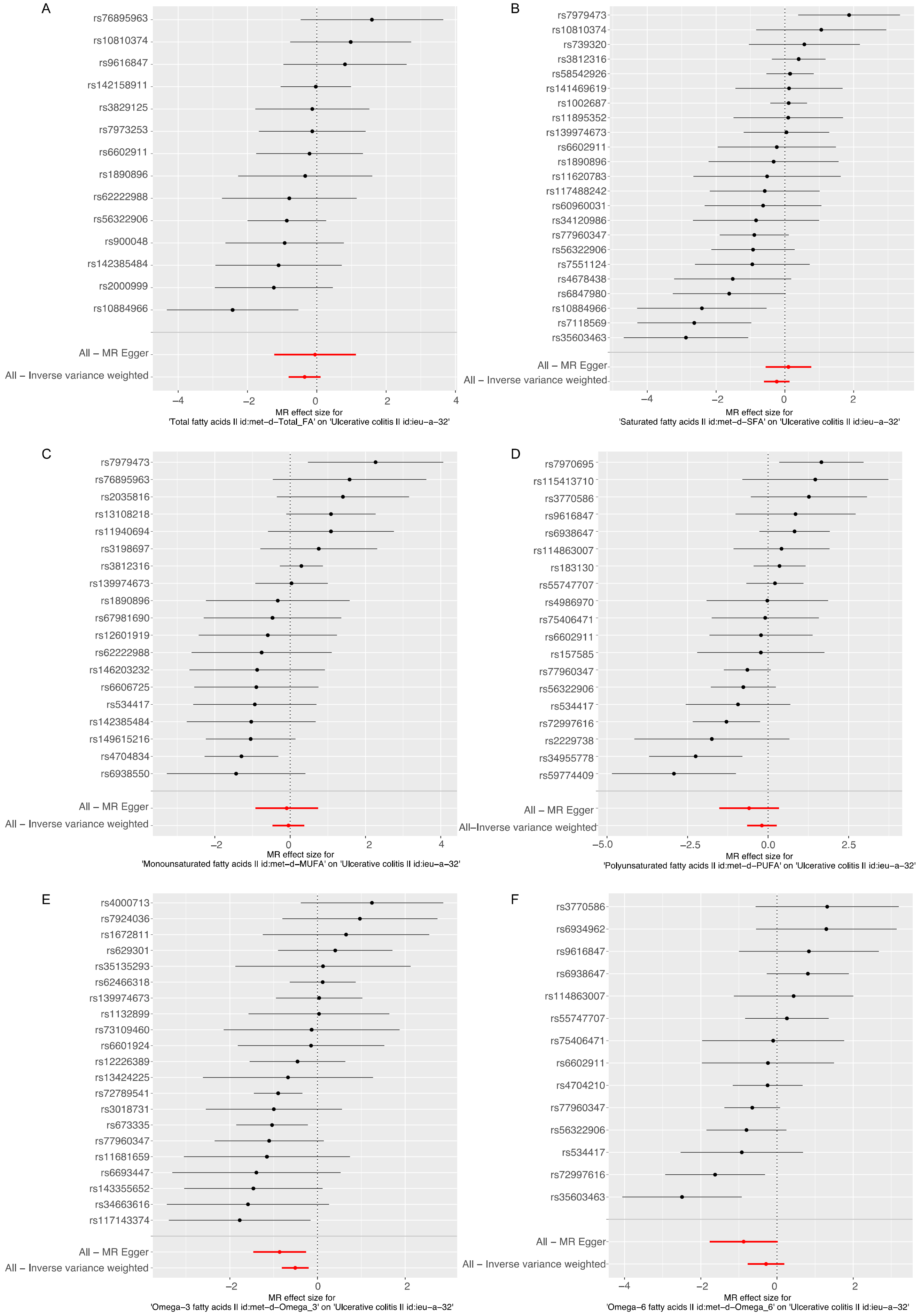

Figure S2. Forest plot of fatty acids and the risk of Ulcerative colitis.

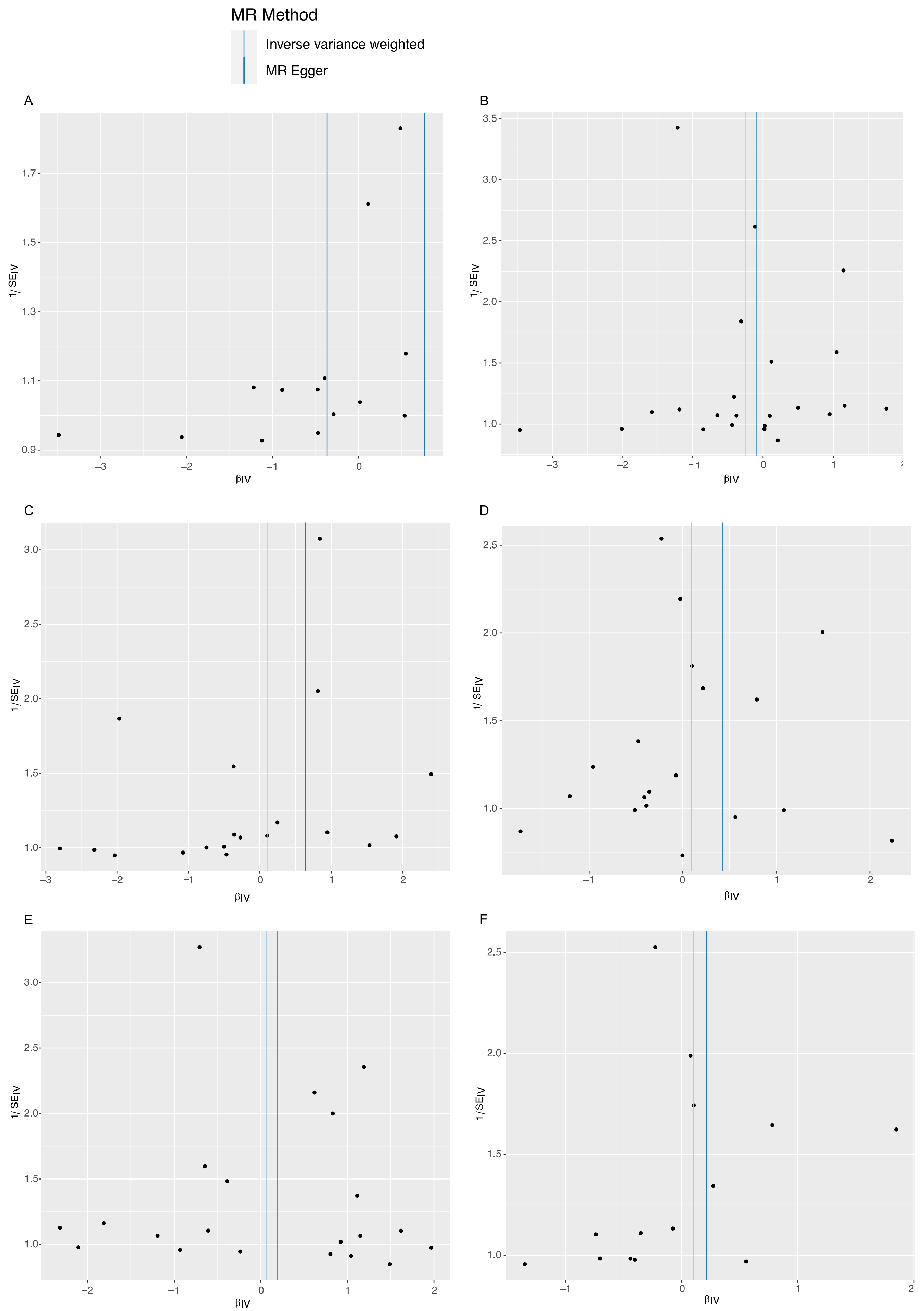

Figure S3. Funnel plot of the inverse variance weighted MR estimate of each fatty acids SNP with Crohn's disease versus 1/SEIV.

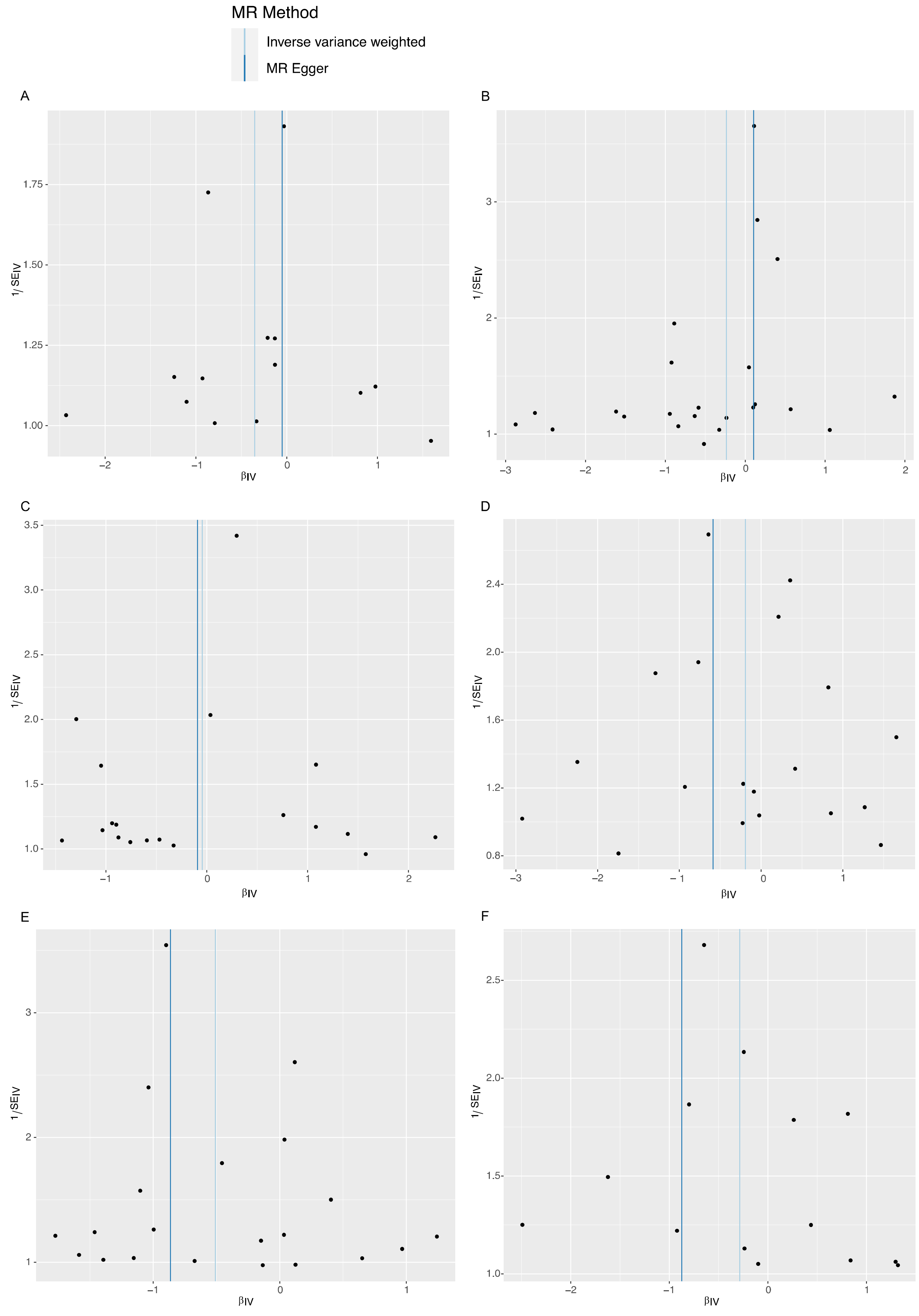

Figure S4. Funnel plot of the inverse variance weighted MR estimate of each fatty acids SNP with Ulcerative colitis versus 1/SEIV.
